# CXCL2/10/12/14 are prognostic biomarkers and correlated with immune infiltration in hepatocellular carcinoma

**DOI:** 10.1042/BSR20204312

**Published:** 2021-06-18

**Authors:** Tong Lin, E Zhang, Pei-pei Mai, Ying-zhao Zhang, Xiang Chen, Li-sheng Peng

**Affiliations:** 1 The Fourth Clinical Medical School, Guangzhou University of Chinese Medicine, Shenzhen, China; 2 Department of Science and Education, Shenzhen Traditional Chinese Medicine Hospital, Shenzhen, China

**Keywords:** chemokines, CXCL, hepatocellular carcinoma, immune infiltration, prognostic biomarker

## Abstract

**Background:** C-x-C motif chemokine ligands (CXCLs) are critical regulators of cancer immunity and angiogenesis, which affect disease progression and treatment responses. The character of each CXCL in the prognosis and immune infiltration of hepatocellular carcinoma (HCC) patients is unclear yet. **Methods:** Differentially expressed CXCLs between HCC and normal control were screened by Oncomine and GEPIA2. Genetic alternations of CXCLs in HCC were analyzed by cBioPortal. Clinicopathological relevance of CXCLs in HCC patients was analyzed using UALCAN. The prognostic value of CXCLs was evaluated using univariate and multivariate analyses. Correlations of CXCLs’ expression with immune infiltration, chemokines and their receptors were assessed integrating TIMER, TISIDB, and GEPIA2. The co-expressed genes of CXCLs were discovered, and functional enrichment analysis was performed for them. **Results:** *CXCL9/10* was significantly higher expressed while *CXCL2/12/14* was lower expressed in HCC than normal tissues, but they didn’t show significant clinicopathological relevance in HCC patients. High-expression of *CXCL2/10/12/14* indicated favorable outcomes of HCC patients. The expression of *CXCL9/10/12/14* was significantly positively correlated with not only the infiltration and biomarkers’ expression of various tumor-infiltrating immune cells but also the abundance of chemokines and their receptors. The co-expressed genes of the five CXCLs were extracellular components and regulated immune or inflammatory responses and signaling pathways of chemokine, Toll-like receptor and tumor necrosis factor might be involved. **Conclusion:** The present study proposed *CXCL2/10/12/14* might predict outcomes of HCC patients and were extensively related with the immune microenvironment in HCC. It would be a prospective therapeutic strategy for HCC to enhance effective immunity surveillance through intervening in these CXCLs.

## Introduction

Hepatocellular carcinoma (HCC) is the sixth most frequently diagnosed and the fourth lethal cancer globally [1]. Due to occult symptoms, a majority of HCC patients are diagnosed at advanced stages when curative surgery is unavailable. Although dramatic achievements have been made in comprehensive treatments, frequent therapy resistance, recurrence, and metastasis lead to the poor prognosis of patients with a 5-year survival rate of about 12% [2]. Over the last 10 years, immunotherapies, especially immune checkpoint inhibitors, have revolutionized the field of cancer. However, the general clinical response of immunotherapy is unsatisfying due to cancer immune escape [3].

Chemokines are small secreted proteins that can induce migration of various cells, including immune cells, epithelial cells, and cancer cells; thus, they are important modulators of cancer immunity, angiogenesis, progression, and therapy [4]. The chemokine superfamily is divided into CC, CXC, CX3C, and XC subfamilies, based on the position of conserved cysteine residues [4]. C-x-C motif chemokine ligand (CXCL), in which the ‘C’s stand for two N-terminal cysteines separated by a random amino acid (‘x’). Sixteen CXCL family members have been identified in human and are named by identifying numbers from CXCL1 to CXCL17, except for CXCL15, which is only reported in mice [5]. CXCLs are further divided into two subgroups, depending on the presence of a Glu-Leu-Arg (ELR) motif at the first conserved cysteine residue. ELR+ CXCLs, including CXCL1-3, CXCL5-8, and CXCL17, bind to CXC chemokine receptor 2 (CXCR2) to promote endothelial cell survival and tumor angiogenesis, whereas most ELR− CXCLs, including CXCL4, CXCL9-11, and CXCL16, bind to CXCR3 to inhibit endothelial cell proliferation and angiogenesis [6].

Immune cells recruited into the tumor microenvironment (TME) by chemokines are called tumor-infiltrating immune cells (TIICs), which play critical roles in the initiation, progression, metastasis, and therapeutic response of cancers [7]. Distinct subsets of TIICs can act at opposite poles. For example, CD8+ T cells and natural killer (NK) cells exert effective anticancer immunity surveillance, whereas regulatory T cells (Tregs) and M2 tumor-associated macrophages (TAMs) foster immunosuppression in HCC [4,8]. Some CXCLs had been reported to promote cancer by stimulating immune-suppressive TIICs. For instance, CXCL16 might induce tumoral phenotypes in solid tumors by stimulating macrophage polarization [9]. By contrast, elevated expression of CXCL10 in tumor cells could increase NK cell infiltration in tumors and prolong NK cell-dependent survival of mice [10]. Therefore, it is a promising strategy to enhance the immunotherapy efficacy by increasing the infiltration of effective TIICs in HCC trough regulating CXCLs [11]. However, the roles of diverse CXCL family members and their interactions with TIICs in HCC are not fully elucidated.

In the present study, we comprehensively analyzed the expression and prognostic values of CXCLs along with their correlations with immune infiltration in HCC patients. Moreover, a co-expression network of CXCLs was constructed, whose biological functions were explored. The findings of our study might provide an overview of CXCLs’ roles in the development and immune microenvironment of HCC.

## Materials and methods

### Analysis of CXCLs’ differential expression between HCC and normal liver samples

Gene Expression Profiling Interactive Analysis 2 (GEPIA2) (http://gepia2.cancer-pku.cn/) is a web portal for analyzing the transcriptome data of 9736 tumors and 8587 normal samples from the Cancer Genome Atlas (TCGA) and the GTEx projects [12]. The expression of CXCL family genes in HCC compared with normal liver samples was analyzed by GEPIA2 using HCC data (*n*=369) from TCGA and normal liver data (*n*=160) combined TCGA and GTEx datasets.

The differential mRNA expression of CXCLs between HCC and adjacent normal tissues was further confirmed using Oncomine (https://www.oncomine.org/) server, which analyzes gene expression integrating data from published literature, the Stanford Microarray Database, and the NCBI Gene Expression Omnibus (GEO) [13]. CXCLs meeting |log_2_(fold change)| > 1 and *P* value < 0.05 were considered significantly differentially expressed between HCC and normal liver tissues. The overlapping CXCLs between the above two databases were included in our following investigations.

### Analysis of genomic alternations of CXCLs in HCC

cBioPortal (http://www.cbioportal.org/) is a comprehensive web resource providing visual and multidimensional cancer genomics data [14,15]. Genomic alternation profiles including mutations, putative copy-number alterations, and mRNA expression were analyzed by cBioPortal using the data of 360 complete HCC samples from ‘TCGA, Firehose Legacy’ dataset.

### Analysis of CXCLs’ expression in HCC patients with distinct clinicopathological features

UALCAN (http://ualcan.path.uab.edu) is an interactive platform for in-depth analysis of cancer omics data from TCGA [16]. Associations between CXCLs’ expression with distinct clinicopathological features of HCC patients, including genders, ages, pathological stages, and tumor grades were analyzed using UALCAN.

### Analysis of the prognostic significance of CXCLs in HCC patients

Kaplan–Meier (KM) Plotter (http://www.kmplot.com/) is an online tool providing gene expression profiles with patients’ survival information of various cancers [17]. KM Plotter was applied to evaluate associations of CXCLs’ expression and overall survival (OS), relapse-free survival (RFS), progression-free survival (PFS), and disease-free survival (DSS) of HCC patients. All cases were split into two groups by the median of a gene’s expression level to conduct univariate analysis.

The prognostic value of CXCLs was also validated using SurvExpress (http://bioinformatica.mty.itesm.mx:8080/Biomatec/SurvivaX.jsp) [18], a web tool providing multivariate survival analysis and risk assessment for a list of genes in human cancer datasets. Here, we used the data from ‘LIHC-TCGA-Liver hepatocellular carcinoma June 2016’ (*n*=361) to perform a multivariate Cox proportional hazards regression. A prognostic risk score was calculated for each HCC patient, and the patients were divided into high- and low-risk groups by the best cutoff of the scores.

### Analysis of correlations between CXCLs’ expression and immune infiltration in HCC

Correlations between CXCLs’ expression and infiltration levels of diverse TIICs, including CD8+ T cells, CD4+ T cells, B cells, neutrophils, macrophages, dendritic cells (DCs), NK cells, and myeloid-derived suppressor cells (MDSCs) in HCC were assessed using Tumor IMmune Estimation Resource (TIMER) (http://timer.cistrome.org) [19]. Correlations between CXCLs’ expression and abundance of subsets of TIICs, chemokines, and chemokine receptors were further explored using TISIDB (http://cis.hku.hk/TISIDB) [20]. The two web portals both facilitate the investigation of tumor-immune interactions covering multiple cancer types. Moreover, correlations between the expression of CXCLs and biomarkers of TIICs were analyzed using GEPIA2.

### Co-expression network of CXCLs and functional enrichment analysis

A co-expression network of CXCLs was constructed using the GeneMANIA plugin of Cytoscape software (Version 3.7.0) [21]. Then, Gene Ontology (GO) and Kyoto Encyclopedia of Genes and Genomes (KEGG) pathway enrichment analyses were performed for all genes in the co-expression network, using Database for Annotation, Visualization, and Integrated Discovery (DAVID) server (https://david.ncifcrf.gov/home.jsp) [22]. GO enrichment analysis annotated the biological functions of genes in three aspects: biological process (BP), cellular component (CC), and molecular function (MF).

### Statistical analysis

The comparison of gene expression levels in HCC and normal tissues in Oncomine and UALCAN was performed using Student’s *t*-test, which by GEPIA2 was conducted using one-way ANOVA test. For KM Plotter database, log-rank test was performed to compare the prognostic difference between two groups and generate hazard ratio (HR), 95% confidence interval (CI), and *P* values. For SurvExpress platform, a Cox proportional hazard regression model was used to evaluate the prognostic value of CXCLs’ signature. Survival curves were generated applying Kaplan–Meier method. Spearman’s method was performed to analyze correlations between a gene’s expression and immune infiltration or chemokines or TIIC biomarkers’ expression. Correlation strength was measured by correlation coefficient (*r*): 0.00–0.19 was ‘very weak’, 0.20–0.39 was ‘weak’, 0.40–0.59 was ‘moderate’, 0.60–0.79 was ‘strong’, and 0.80–1.0 is ‘very strong’ [23,24]. For all two-tailed analyses, *P* values < 0.05 were considered statistically significant, and false discovery rates (FDRs) < 0.05 were additional criteria for functional enrichment analyses.

## Results

### Differentially expressed CXCLs between HCC and normal liver samples

**Figure 1:**
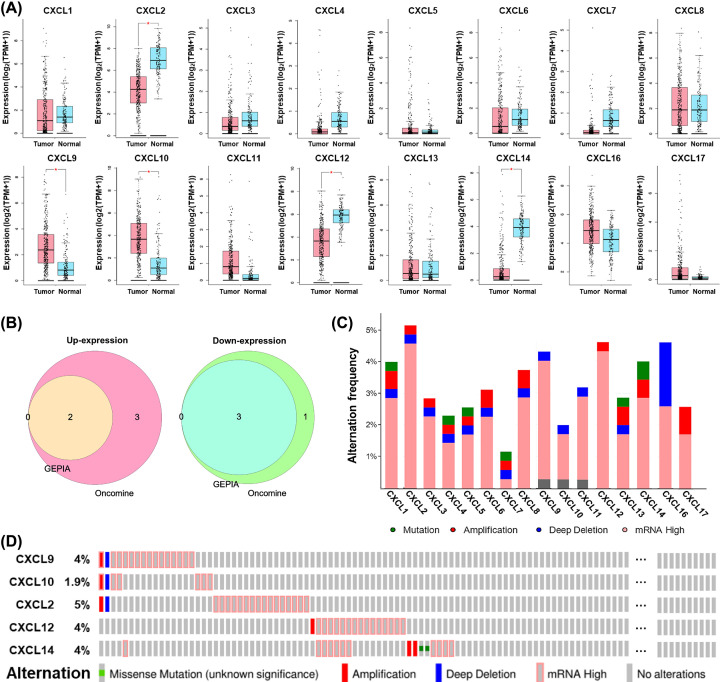
Expression and alternations of CXCL family genes in HCC (**A**) The significant differential expression of CXCL family genes between HCC (*n* = 369) and normal liver tissues (*n* = 160) (GEPIA). *log_2_(fold change)| > 1 and *P* value < 0.05; TPM, transcript per million. (**B**) Venn diagrams show the overlapping up-expressed and down-expressed CXCLs between Oncomine and GEPIA databases. (**C**) The alternation frequency of CXCL family members in HCC (*n* = 360). (**D**) An overview of the alternations occurring in *CXCL9/10/2/12/14* in HCC samples (cBioPortal).

First, the differential expression of CXCL family genes between HCC and normal liver tissues was analyzed integrating GEPIA2 and Oncomine databases. The results from GEPIA2 database showed *CXCL9/10* expression was significantly higher, whereas *CXCL2/12/14* was lower in HCC, compared with normal liver tissues (Figure 1A). In Oncomine, *CXCL6/8/9/10/11* was significantly up-expressed in HCC in one, one, one, two, and one datasets, while *CXCL1/2/12/14* was down-expressed in HCC in one, five, four, and four datasets, respectively, versus adjacent normal tissues (Table 1). Therefore, *CXCL9/10* was consistently significantly up-expressed, whereas *CXCL2/12/14* was down-expressed in HCC versus normal controls in the two databases (Figure 1B), the five CXCLs were included in our further study.

**Table 1:** The significantly differentially expressed CXCLs between HCC and normal liver tissues (Oncomine)

Gene name	Fold change	*P* value	Sample size		Reference (PMID)
			HCC	Normal	
*CXCL1*	-4.16	1.76E-20	102	72	12058060
	-6.54	8.33E-60	225	220	21159642
	-3.60	4.65E-17	102	72	12058060
*CXCL2*	-5.74	1.45E-07	35	10	17393520
	-7.40	2.59E-07	225	220	21159642
	-2.27	9.36E-07	19	38	19098997
*CXCL6*	3.75	2.15E-07	19	38	19098997
*CXCL8*	3.32	7.84E-06	19	38	19098997
*CXCL9*	5.33	6.20E-17	19	38	19098997
*CXCL10*	9.27	6.33E-17	19	38	19098997
	5.93	1.78E-04	35	10	17393520
*CXCL11*	2.19	2.26E-06	19	38	19098997
*CXCL12*	-5.34	2.32E-93	225	220	21159642
	-2.39	4.32E-31	102	72	12058060
	-4.04	4.43E-11	225	220	21159642
	-4.23	3.82E-09	35	10	17393520
*CXCL14*	-10.94	6.98E-154	225	220	21159642
	-9.67	1.24E-21	225	220	21159642
	-12.90	1.96E-43	102	72	12058060
	-13.98	6.81E-10	35	10	17393520

Note: The screening threshold was set as: data type of mRNA, |fold change| of 2.0, *P* value of 0.05, and the rank of top 10% gene; PMID, PubMed Unique Identifier.

### Genetic alternations of CXCLs in HCC

Following, genetic alternations of CXCL family in HCC patients were analyzed using cBioPortal. Overall, missense mutation, putative copy-number alterations including amplification and deep deletion, together with mRNA overexpression were detected in a total of 114 out of 360 (32%) HCC samples, and mRNA overexpression was the most frequent alteration (Figure 1C). To be specific, 16 (4%), 7 (1.9%), 18 (5%), 16 (4%), and 15 (4%) patients were observed with alternations of *CXCL9, CXCL10, CXCL2, CXCL12*, and *CXCL14*, respectively (Figure 1D).

### Clinicopathological relevance of CXCLs

**Figure 2:**
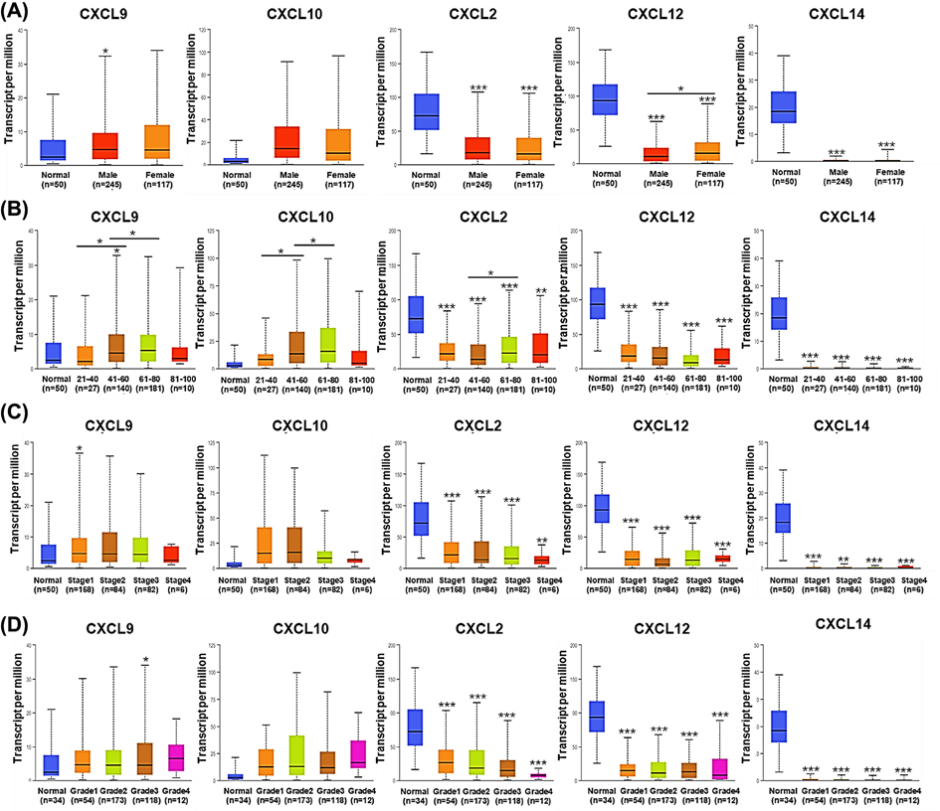
Expression of the five CXCLs in HCC patients with distinct clinicopathological parameters (UALCAN) Expression of CXCLs in HCC patients classified by (**A**) genders, (**B**) ages, (**C**) pathological stages, and (**D**) tumor grades. (*P<0.05, **P<0.01, ***P<0.001).

Associations of the expression of the five CXCLs with clinicopathological characteristics of HCC patients were investigated using UALCAN. In the aspect of genders, *CXCL12* was expressed higher in females (*P*<0.05) than males (Figure 2A). Speaking of ages, middle-aged and senile HCC patients (41–80 years old) tended to have higher expression levels of *CXCL9/10*, compared with those in youth (21–40 years old) (*P*<0.05) (Figure 2B). However, the expression of the five CXCLs showed no significant difference among diverse pathological stages and tumor grades (Figure 2C,D).

### Prognostic significance of CXCLs in HCC patients

**Figure 3:**
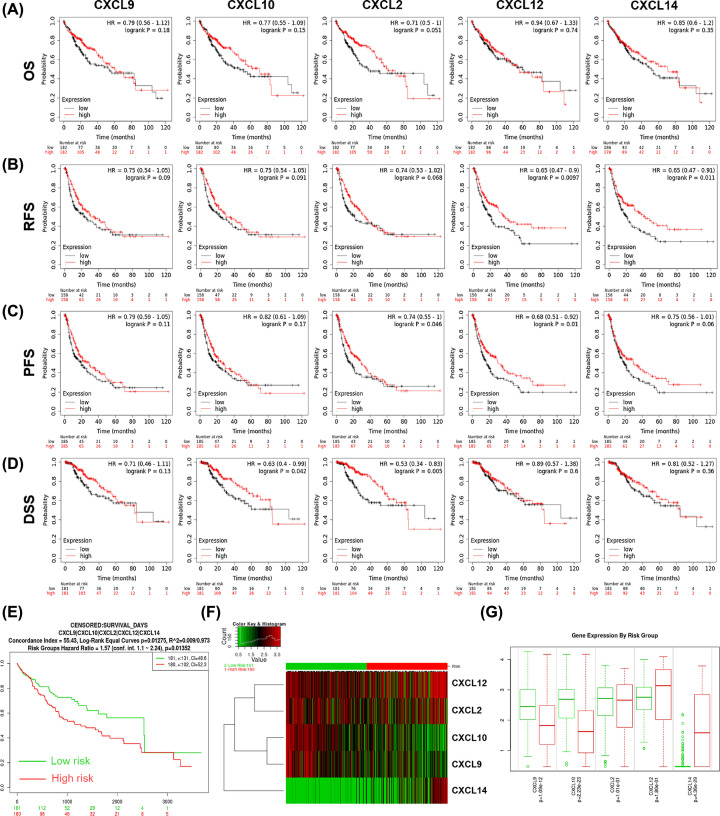
Prognostic significance of the five CXCLs in HCC patients The survival curves showed the associations between the expression of CXCLs with (**A**) OS, (**B**) RFS, (**C**) PFS, and (**D**) DDS of HCC patients (KM Plotter). (**E**-**G**) The prognostic value of CXCLs’ signature in HCC (SurvExpress). (**E**) Survival curves of the low- (green, *n* = 181) and high- (red, *n* = 180) risk groups. (**F**) A heat map showing the clustered expression of CXCLs between the low- and high-risk groups. (**G**) Comparison of the expression of each CXCL member between low- and high-risk groups. OS, overall survival; RFS, relapse-free survival; PFS, progression-free survival; DDS, disease-free survival; HR, hazard ratio; CI, confidence interval.

Wondering the prognostic significance of the five CXCLs in HCC patients, survival analysis was performed using KM Plotter. As shown in Figure 3A–D, higher expression of *CXCL10* was associated with better DSS (HR = 0.63, *P*=0.042); overexpression of *CXCL2* was associated with longer PFS and DSS (PFS: HR = 0.74, *P*=0.046; DDS: HR = 0.67, *P*=0.016); up-expression of *CXCL12* was related to favorable RFS and PFS (RFS: HR = 0.65, *P*=0.0097; PFS: HR = 0.68, *P*=0.01); and up-regulation of *CXCL14* was linked to better RFS (HR = 0.65, *P*=0.011) of HCC patients.

In addition, the results from SurExpress showed the low-risk group displayed significant favorable OS compared with the high-risk group (HR = 1.57, 95% CI = 1.1-2.24, *P*=0.0135) (Figure 3E). In the high-risk group, the expression of *CXCL9* (*P*=1.09E-12) and *CXCL10* (*P*=2.23E-23) was significantly lower, while *CXCL14* (*P*=4.36E-29) was higher than that in the low-risk group (Figure 3 F,G).

### Correlations between CXCLs’ expression and immune infiltration in HCC

**Figure 4:**
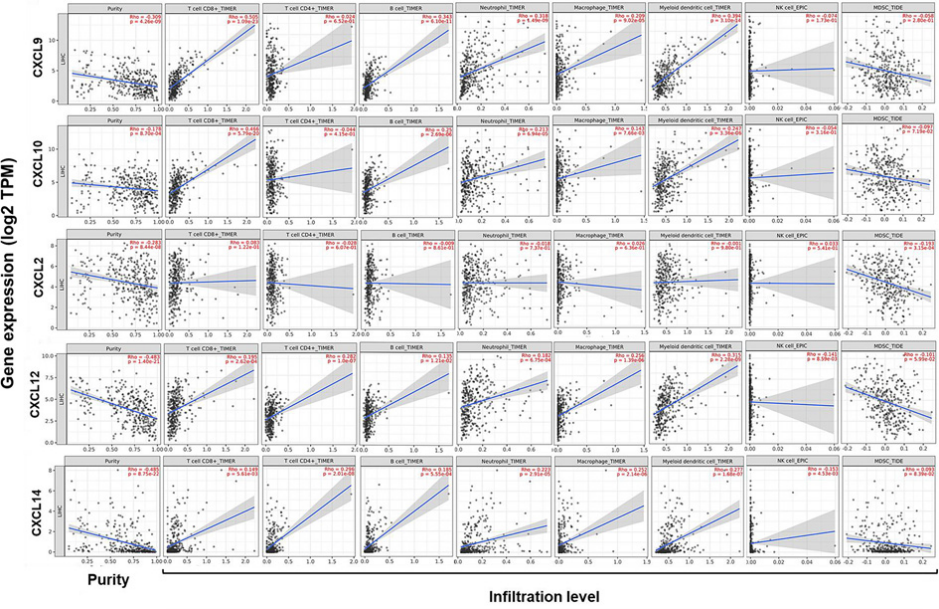
Correlations between CXCLs’ expression with the immune infiltration in HCC (TIMER) Correlations between CXCLs’ expression with tumor purity, and infiltration levels of CD8+ T cells, CD4+ T cells, B cells, neutrophils, macrophages, DCs, NK cells, and MDSCs in HCC. DCs, dendritic cells; NK cells, natural killer cells; MDSCs, myeloid-derived suppressor cells, Th, helper T cell.

Subsequently, correlations between the five CXCLs’ expression and immune infiltration in HCC were investigated combined TIMER and TISIDB databases. Tumor purity is defined as the proportion of cancer cells in tumor admixture, which influence the evaluation of immune infiltration. All analyses about immune infiltration were adjusted with the corresponding tumor purity in the present study [25]. As presented in Figure 4, the five CXCLs’ expression was consistently negatively correlated to the tumor purity.

According to the results from TIMER, *CXCL9/10/12/14* expression was positively correlated with the infiltration of CD8+ T cells, B cells, neutrophils, macrophages, and DCs (*P* ≤ 8.70E-04). *CXCL12/14* expression was positively correlated with the infiltration of CD4+ T cells but negatively correlated with that of NK cells (*P* ≤ 8.59E-03). *CXCL2* expression presented a negative correlation with the infiltration of MDSCs (*P* = 3.15E-04). Noteworthy, correlation strength of the infiltration of CD8+ T cells with the expression of *CXCL9* (*r*=0.505, *P*=1.09E-23) and *CXCL10* (*r*=0.466, *P*=5.79E-20) was moderate (Figure 4).

**Figure 5:**
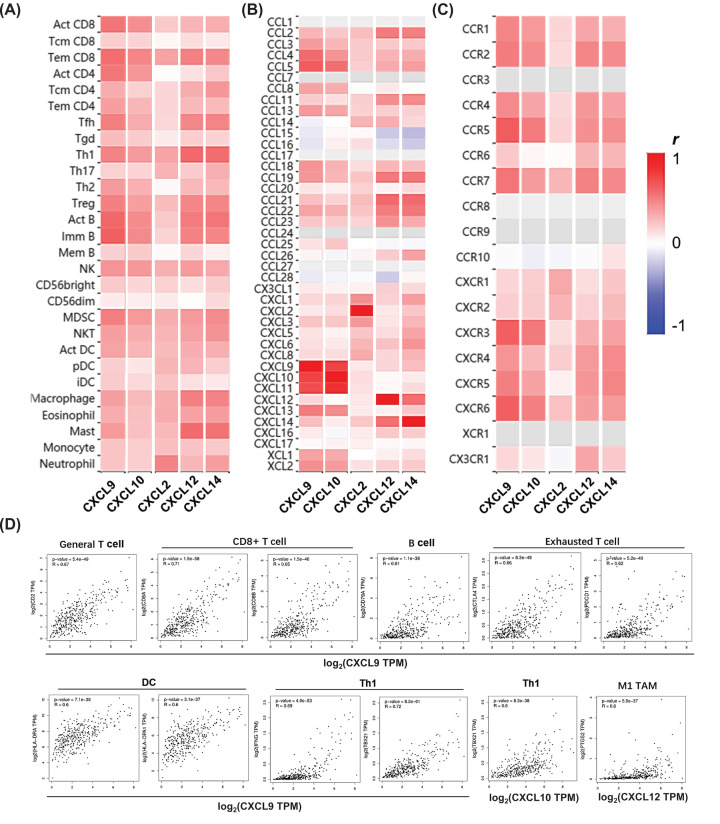
Correlations between CXCLs’ expression with the abundance of TIICs, chemokines, chemokine receptors, and biomarkers’ expression of some TIICs in HCC Correlations between CXCLs’ expression with (**A**) the abundance of TIIC subsets, (**B**) chemokines, and (**C**) chemokine receptors (TISIDB). (**D**) Strong correlations between the expression of *CXCL9/10/12* and biomarkers of T cells, CD8+ T cells, B cells, exhausted T cells, DCs, Th1 cells, and M1 TAMs in HCC (GEPIA). “Act”, “Tcm”, and “Tem” CD8/CD4 represent activated, central memory, and effector memory CD8+/CD4+ T cells respectively. Tfh, follicular helper T cell; Tgd, gamma delta T cells. “Act”, “Imm”, and “Mem” B represent activated, immune, and memory B cells respectively. “CD56bright” and “CD56dim” mean CD56bright and CD56dim natural killer cells respectively. NKT, natural killer T cell. “Act DC”, “pDC”, and “iDC” mean activated, plasmacytoid, and immune dendritic cells respectively.

As exhibited in Figure 5A, the expression of these CXCLs was generally positively correlated with the abundance of 28 kinds of TIICs in TISIDB. Consistent with the findings from TIMER, *CXCL9/10/12/14* expression showed moderate to strong positive correlations with the abundance of effector memory CD8+ T cells, along with activated and immature B cells. Moderate positive correlations were also found between *CXCL9/10* expression and the abundance of activated CD8+ T cells; *CXCL9/12/14* expression and the abundance of follicular helper T cell (Tfh), type-1 helper T cell (Th1), and Tregs; *CXCL12/14* expression and the abundance of macrophages and mast cells; *CXCL2* expression and the abundance of neutrophils.

As for relations among CXCLs and other chemokines and their receptors, *CXCL9-11* highly interacted with each other, *CXCL12/14* connected with a moderate degree. Positive connections with moderate to strong strength were observed between *CXCL9/10* and *CCL4/5*, also *CXCL12/14* and *CCL2/11/19/21/22/23* (Figure 5B). Moderate to strong correlations were found in *CXCL9/10/12/14* with *CCR1/2/4/5/7* also *CXCR3/4/5/6* (Figure 5C).

### Correlations between the expression of CXCLs and biomarkers of TIICs in HCC

To confirm the participation of TIICs, correlations between the expression of CXCLs and biomarkers of TIICs in HCC were further analyzed using GEPIA2. The biomarkers of all TIICs mentioned above were investigated, together with monocytes, M1/M2 TAMs, and subsets of T cells, including Th1, Th2, Th17, Tfh, Tregs, and exhausted T cells. A little bit different from the results from TIMER, it showed that *CXCL9/10/12/14* expression was not only positively correlated with the expression of almost all biomarkers of B cells, CD8+ T cells, neutrophils, TAMs, and DCs, but also subsets of CD4+T cells, monocytes, and NK cells, implying the potential involvement of these TIICs (*P*<0.05) (Table 2). Notably, strongly positive correlations were observed between the expression of *CXCL9* and signatures of B cells (CD79A), general T cells (CD2), Th1 cells (TBX21 and IFNG), CD8+ T cells (CD8A/B), exhausted T cells (PDCD1 and CTLA4), and DCs (HLA-DRA and HLA-DPA1) (*P*<3.1E-37); *CXCL10* and Th1 (TBX21), as well as *CXCL12* and M1 TAMs (PTGS2) (*P*<5.8E-37) (Figure 5D). Till now, the above findings indicated CXCLs' regulations on immune infiltrate of various TIICs in HCC.

**Table 2:** Correlations of the expression of CXCLs and biomarkers of TIICs in HCC (GEPIA)

Types of TIICs	Gene markers	CXCL9		CXCL10		CXCL2		CXCL12		CXCL14	
		*r*	*P*	*r*	*P*	*r*	*P*	*r*	*P*	*R*	*P*
B cell	CD19	0.4	**1.6E−15**	0.3	**7.3E−09**	0.033	0.53	0.32	**2.2E−10**	0.41	**1.2E−16**
	CD79A	0.61	**1.1E−38**	0.42	**5.0E−17**	0.11	**0.03**	0.5	**3E−24**	0.52	**6.4E−27**
T cell (general)	CD3D	0.56	**1.8E−32**	0.44	**1.7E−18**	0.098	0.059	0.25	**8.7E−07**	0.4	**2.6E−15**
	CD2	0.67	**5.4E−49**	0.53	**1.9E−28**	0.19	**0.00035**	0.44	**3E−19**	0.47	**8E−22**
Th1	TBX21	0.72	**8.2E−61**	0.6	**8.3E−38**	0.18	**0.00073**	0.42	**5.4E−17**	0.35	**6.3E−12**
	STAT4	0.37	**1.4E−13**	0.4	**2.7E−15**	0.27	**2.3E−07**	0.32	**2.5E−10**	0.32	**3.5E−10**
	STAT1	0.57	**5.9E−33**	0.56	**1E−31**	0.059	0.26	0.32	**6E−10**	0.25	**1.3E−06**
	TNF	0.51	**7.4E−26**	0.37	**1.9E−13**	0.15	**0.0046**	0.38	**8.5E−14**	0.38	**2.3E−14**
	IFNG	0.69	**4.9E−53**	0.55	**2.5E−30**	0.016	0.76	0.15	**0.0029**	0.26	**4.7E−07**
Th2	GATA3	0.53	**5.9E−28**	0.37	**1.4E−13**	0.19	**0.00017**	0.49	**1.7E−23**	0.53	**3.3E−28**
	STAT6	0.15	**0.0029**	0.14	**0.0065**	0.11	**0.03**	0.12	**0.024**	0.098	0.059
	IL13	0.19	**0.00019**	0.16	**0.0022**	0.025	0.63	0.029	0.58	0.13	**0.013**
	STAT5A	0.39	**4.5E−15**	0.36	**1.8E−12**	0.088	0.091	0.3	**4.2E−09**	0.29	**1.1E−08**
Tfh	BCL6	0.23	**6.4E−06**	0.18	**0.00038**	0.076	0.14	0.084	0.11	0.045	0.39
	IL21	0.36	**1.4E−12**	0.29	**9.4E−09**	−0.066	0.2	0.11	**0.03**	0.14	**0.0087**
Th17	STAT3	0.22	**1.3E−05**	0.18	**0.00059**	0.33	**8.9E−11**	0.26	**3.1E−07**	0.23	**9.8E−06**
	IL17A	0.066	0.21	0.11	**0.035**	0.076	0.15	0.033	0.52	0.035	0.51
Treg	FOXP3	0.48	**3.3E−22**	0.46	**6.4E−21**	0.13	**0.014**	0.15	**0.0049**	0.19	**0.00017**
	CCR8	0.55	**2.2E−30**	0.47	**1.1E−21**	0.18	**0.00036**	0.32	**3.6E−10**	0.31	**2.1E−09**
	TGFB1	0.32	**2.3E−10**	0.13	**0.015**	0.075	0.15	0.48	**3.9E−23**	0.51	**3.6E−26**
CD8+ T	CD8A	0.71	**1.5E−58**	0.54	**1.2E−29**	0.16	**0.0024**	0.44	**1.6E−18**	0.42	**1.9E−17**
	CD8B	0.65	**1.5E−46**	0.49	**1.4E−23**	0.11	**0.043**	0.35	**2.4E−12**	0.39	**1E−14**
Exhausted T cell	PDCD1	0.62	**5.2E−40**	0.42	**1.4E−17**	0.058	0.27	0.37	**3.9E−13**	0.4	**1.3E−15**
	CTLA4	0.66	**8.3E−48**	0.47	**6.6E−22**	0.15	**0.0035**	0.24	**3.4E−06**	0.35	**2.3E−12**
	LAG3	0.48	**3.8E−23**	0.38	**6.8E−14**	−0.017	0.75	0.15	**0.0035**	0.24	**3.3E−06**
	TIM3	0.54	**5.5E−29**	0.4	**9.5E−16**	0.18	**0.00052**	0.42	**2.6E−17**	0.47	**2.7E−21**
	GZMB	0.58	**1.1E−34**	0.42	**2.2E−17**	0.094	0.072	0.23	**9.5E−06**	0.28	**3.5E−08**
NK cell	KIR2DL1	0.26	**2.8E−07**	0.19	**0.00033**	0.15	**0.0036**	0.12	**0.023**	0.12	**0.022**
	KIR2DL3	0.4	**1.6E−15**	0.31	**8.7E−10**	0.045	0.82	0.16	**0.002**	0.19	**0.00033**
	KIR3DL1	0.27	**1.9E−07**	0.25	**1.3E−06**	0.13	**0.015**	0.13	**0.01**	0.052	0.32
	KIR3DL2	0.43	**2.3E−18**	0.3	**4E−09**	0.024	0.65	0.29	**2.1E−08**	0.27	**1E−07**
	KIR3DL3	0.16	**0.0019**	0.13	**0.012**	−0.012	0.82	−0.01	0.84	0.073	0.16
	KIR2DS4	0.26	**3E−07**	0.22	**1.4E−05**	0.062	0.24	0.16	**0.0025**	0.083	0.11
Neutrophil	CD11b	0.32	**4.8E−10**	0.35	**4.9E−12**	0.22	**1.6E−05**	0.24	**1.9E−06**	0.28	**7.5E−08**
	CCR7	0.57	**7.4E−33**	0.42	**1.4E−17**	0.29	**1.2E−08**	0.55	**3.7E−30**	0.5	**1.6E−24**
	CD66b	0.081	0.12	0.036	0.49	0.046	0.38	0.033	0.53	0.055	0.29
M1 TAM	NOS2	0.13	**0.016**	0.13	**0.015**	0.11	**0.039**	0.25	**1.2E−06**	0.14	**0.0053**
	PTGS2	0.3	**2.6E−09**	0.19	**0.00018**	0.34	**2.2E−11**	0.6	**5.8E−37**	0.53	**3.7E−28**
	IRF5	0.18	**0.00061**	0.19	**0.00024**	0.035	0.5	0.078	0.13	0.12	**0.017**
M2 TAM	CD163	0.43	**1.2E−17**	0.39	**3.8E−15**	0.24	**2.2E−06**	0.42	**5.3E−17**	0.4	**7.3E−16**
	VSIG4	0.38	**5.9E−14**	0.36	**8.4E−13**	0.28	**5.2E−08**	0.42	**2.3E−17**	0.4	**2.6E−15**
	MS4A4A	0.48	**6.8E−23**	0.42	**3.4E−17**	0.28	**7.8E−08**	0.45	**1.4E−19**	0.34	**2.6E−11**
TAM	CCL2	0.37	**4.2E−13**	0.3	**6.9E−09**	0.27	**1.7E−07**	0.58	**6.8E−35**	0.54	**2.3E−29**
	CD68	0.37	**3.0E−13**	0.28	**4.5E−08**	0.11	**0.037**	0.37	**1.4E−13**	0.26	**2.5E−07**
	IL10	0.46	**3.5E−21**	0.35	**4.4E−12**	0.13	**0.016**	0.37	**1E−13**	0.29	**1.9E−08**
Monocyte	CD86	0.58	**9.5E−35**	0.46	**1.2E−20**	0.18	**0.00038**	0.48	**3.5E−22**	0.45	**5E−20**
	CD115	0.49	**3.7E−24**	0.4	**1.1E−15**	0.23	**5.6E−06**	0.53	**8.7E−28**	0.46	**1.3E−20**
DC	HLA-DPB1	0.57	**1.6E−33**	0.47	**3E−21**	0.23	**7.1E−06**	0.53	**1.3E−27**	0.47	**9.3E−22**
	HLA-DRA	0.6	**7.1E−38**	0.5	**6.1E−25**	0.28	**7.5E−08**	0.49	**8.7E−24**	0.43	**2.8E−18**
	HLA-DQB1	0.43	**6.8E−18**	0.3	**5E−09**	0.13	**0.013**	0.28	**3.3E−08**	0.32	**4.6E−10**
	HLA-DPA1	0.6	**3.1E−37**	0.5	**3.3E−24**	0.27	**9.4E−08**	0.5	**6.7E−25**	0.43	**6.9E−18**
	CD1C	0.38	**2.1E−14**	0.31	**1.1E−09**	0.2	**8.8E−05**	0.57	**1.8E−33**	0.45	**1.3E−19**
	NRP1	0.24	**2E−06**	0.14	**0.0058**	0.071	0.18	0.33	**4.9E−11**	0.28	**3.3E−08**
	CD11c	0.51	**1.3E−25**	0.41	**1.1E−16**	0.22	**1.3E−05**	0.4	**2.4E−15**	0.41	**3.8E−16**

Note: The correlation analysis was adjusted for the tumor purity. *P* values with statistical significance are shown in bold. TAM, tumor-associated macrophage; Tfh, follicular helper T cell; Th, helper T cell; Treg, regulatory T cell; NK cell, natural killer cell; *r*, the correlation coefficient of Spearman’s analysis.

### Functions of the co-expression network of CXCLs

**Figure 6:**
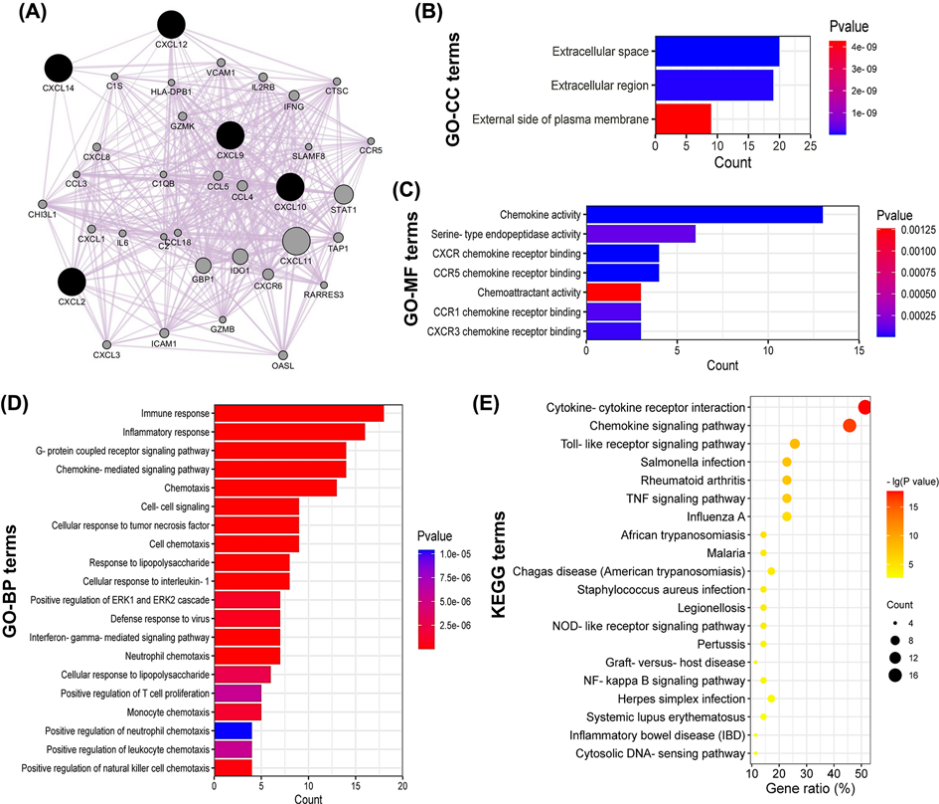
Functions of the co-expression network of CXCLs (**A**) The co-expression network of the five CXCLs. (**B**) All GO-CC terms, (**C**) all GO-MF terms, (**D**) the top 20 GO-BP terms, and (**E**) the top 20 KEGG pathway terms enriched for the co-expressed genes.

To understand the functional mechanisms of the five CXCLs, functional enrichment analysis was performed for the co-expression network of CXCLs. Thirty co-expressed genes of the five CXCLs were identified, so the co-expression network was composed of totally 35 genes (Figure 6A). And 15 genes out of them were CXCLs or C-C motif chemokine ligands (CCLs) or their receptors.

Subsequently, functional enrichment analysis was conducted for all genes in the co-expression network using DAVID platform. The significantly enriched GO-CC (Figure 6B), GO-MF (Figure 6C) and GO-BP (Figure 6D) terms elucidated the co-expressed genes were components of the extracellular region and participated in chemokine-mediated immune or inflammatory responses, as well as G-protein coupled receptor activity through binding with the corresponding chemokine receptors. Moreover, the enriched KEGG pathway terms elucidated these genes mainly involved in cytokine–cytokine receptor interaction and signaling pathways of chemokine, Toll-like receptor, tumor necrosis factor (TNF), and nucleotide-binding oligomerization domain (NOD)-like receptor (NLR), etc. (Figure 6E).

## Discussion

To begin with, five significantly differentially expressed CXCLs overlapping between GEPIA2 and Oncomine databases were screened out, among which *CXCL 9/10* was elevated, while *CXCL2/12/14* was decreased in HCC versus normal control. Interestingly, although mRNA overexpression was the most frequent alternation of *CXCL2/12/14*, they were still low-expressed in HCC. Different from two recent bioinformatics studies, in which *CXCL1/3/5/8* overexpression was reported as unfavorable prognostic indicators of HCC [26,27]. We found high expression of *CXCL2/10/12/14* implied favorable survivals of HCC patients using univariate analysis, even though their expression was not significantly associated with pathological stages and histological grades of HCC patients. In addition, *CXCL9/10* up-regulation indicated lower risk in the multivariate analysis. However, the results on *CXCL14* were contradictory, since low-risk HCC patients presented extremely low *CXCL14* expression.

After that, we observed the five CXCLs’ expression was uniformly negatively correlated to the tumor purity, suggesting their expression might be mainly from immune cells or stromal cells in the TME rather than cancer cells. Generally speaking, *CXCL9/10/12/14* was conformably positively correlated with the infiltration and (or) biomarkers’ expression of diverse TIICs, including CD8+ T cells, B cells, neutrophils, TAMs, DCs, and NK cells. In particular, *CXCL9/10* expression showed moderate correlations with the infiltration of activated CD8+ T cells. *CXCL9/12/14* expression presented quite close relationships with the abundance of subsets of CD4+ T cells, especially Th1 cells. Besides, the expression of *CXCL9/10/12/14* was correlated with *CCR1/2/4/5/7* and *CXCR3/4/5/6* with moderate to strong extents.

It is acknowledged that CD8+ cytotoxic T cells and NK cells are the main undertakers of anticancer immunity in the TME, both can be stimulated by pro-inflammatory cytokines secreted by Th1 cells. A higher density of CD8+ T cells and NK cells in tumor tissues could predict improved treatment responses and the prognosis of HCC patients [28,29]. Consistent with our results, CXCL9/10 can recruit CD8+ T cells, Th1 cells, and NK cells by binding to the common receptor CXCR3 [10,30]. A retrospective study showed HCC patients with higher serum CXCL9 levels had better survivals under sorafenib therapy [31]. And the down-regulation of CXCL9/CXCR3 axis might contribute to HCC recurrence after partial hepatectomy, since it remarkably reduced the proportion of intrahepatic NK cells [32]. DCs are the most potent antigen-presenting cells to motivate effective immune responses of T cells and NK cells once activated by antigens [33,34]. The role of tumor-infiltrating B cells in HCC remains controversial, since it seems to work dually depending to the secretion of inflammatory factors [35]. It was ever reported that the proximity between T cells and B cells indicated a functional interaction that might lead to a better prognosis [36]. Nevertheless, it was also demonstrated that CXCL9-11 would induce the M2-type polarization of TAMs by binding to CXCR3+ B cells, which were correlated with early recurrence of HCC [37]. Overall, the up-regulation of *CXCL9/10* could enhance immune response and the subsequent apoptosis to augment immunotherapy effects [38,39].

MDSCs exert tumor-promoting and immunosuppressive roles in cancers through inducing differentiation and expansion of Tregs, inhibiting DCs, NK cells, and T cells [40–42]. In accordance with our findings, the earlier studies expounded CXCL2/14 were both stably down-regulated in HCC specimens compared with adjacent normal tissues, and whose overexpression might profoundly inhibit angiogenesis and aggressiveness of HCC cells, partly through apoptosis pathways [43,44]. Our results showed *CXCL2* expression was negative correlated with the infiltration of MDSCs, suggesting CXCL2 might ameliorate host immunosurveillance by reducing MDSC generation [45,46]. It had been ever proved that *CXCL14* overexpression could attract effective DCs, NK cells, and T cells [47]. CXCL12 is a common ligand of CXCR4 and CXCR7, which was expressed at significantly lower levels in HCC compared with adjacent normal liver tissues. Controversial to our observations, CXCL12/CXCR4 axis was reported to promote angiogenesis and aggressiveness of HCC cells and facilitate immune escape via inducing MDSCs and plasmacytoid DCs [48–51]. However, the activation of CXCL12/CXCR7 did not affect the prognosis of HCC patients [52]. Hereto, we could deduce the regulations of CXCLs to the tumor immune microenvironment might partly explain their influence on HCC patients’ prognosis. The up-regulation of *CXCL9/10/12/14* might improve outcomes of HCC patients through reinforcing immune surveillance of CD8+ T cells, NK cells, DCs, and Th1 cells, etc., while *CXCL2* might mainly be through reducing MDSCs. It appeared that CXCL12 functioned dually in HCC according to the receptors to which it bound, thus, more explorations are still required.

Finally, a co-expression network of the five CXCLs was constructed and functional enrichment analysis was performed for these co-expressed genes to elucidate their biological functions. The enrichment analysis uncovered these co-expressed genes were components of the external cellular region, and were responsible for chemokine-mediated immune or inflammatory responses, along with G-protein coupled receptor activity. And the signaling pathways of chemokine, Toll-like receptor, TNF, and NLR were involved. All these pathways play vital roles in the inflammation engaged in cancer malignant progress [35,42].

## Conclusion

The present study proposed that elevated *CXCL2/10/12/14* expression might serve as favorable prognostic indicators of HCC patients. The critical regulatory mechanisms might lie in their beneficial modulations of multiple TIICs in the TME, particularly CD8+ T cells, NK cells, DCs, Th1 cells, and MDSCs. The inflammation-related signaling pathways of chemokine, Toll-like receptor, TNF, and NLR were potentially involved. Therefore, it’s enlightened the five CXCLs might exert as possible therapeutic targets to regulate anticancer immunity in the TME of HCC to improve the efficacy of immunotherapy. However, further validated experiments and clinical studies are still required.

## Data Availability

All the data that support the findings of this study are publicly available in: https://www.oncomine.org/, http://gepia2.cancer-pku.cn/, http://www.cbioportal.org/, http://ualcan.path.uab.edu, http://www.kmplot.com/, http://bioinformatica.mty.itesm.mx:8080/Biomatec/SurvivaX.jsp, http://timer.cistrome.org, http://cis.hku.hk/TISIDB, and https://david.ncifcrf.gov/home.jsp.

## Abbreviations

CI: confidence interval
CXCL: C-x-C motif chemokine ligand
HCC: hepatocellular carcinoma
HR: hazard ratio
NLR: NOD-like receptor
NOD: nucleotide-binding oligomerization domain
TIICs: tumor-infiltrating immune cells
TNF: tumor necrosis factor
TME: tumor microenvironment
